# The Impact of Radiotherapy Dose in Patients with Locally Advanced Esophageal Squamous Cell Carcinoma Receiving Preoperative Chemoradiotherapy

**DOI:** 10.3390/curroncol28020129

**Published:** 2021-03-29

**Authors:** Chien-Ming Lo, Yu-Ming Wang, Yen-Hao Chen, Fu-Min Fang, Shun-Chen Huang, Hung-I Lu, Shau-Hsuan Li

**Affiliations:** 1 Department of Thoracic & Cardiovascular Surgery, Kaohsiung Chang Gung Memorial Hospital, Chang Gung University Colledge of Medicine, Kaohsiung 833401, Taiwan; t123207424@cgmh.org.tw; 2 Department of Radiation Oncology, Kaohsiung Chang Gung Memorial Hospital, Chang Gung University Colledge of Medicine, Kaohsiung 833401, Taiwan; scorpion@cgmh.org.tw (Y.-M.W.); fang2569@cgmh.org.tw (F.-M.F.); 3 Department of Hematology-Oncology, Kaohsiung Chang Gung Memorial Hospital, Chang Gung University Colledge of Medicine, Kaohsiung 833401, Taiwan; alex2999@cgmh.org.tw; 4 Department of Pathology, Kaohsiung Chang Gung Memorial Hospital, Chang Gung University Colledge of Medicine, Kaohsiung 833401, Taiwan; shuang@cgmh.org.tw

**Keywords:** esophageal squamous cell carcinoma, chemoradiotherapy, esophagectomy, radiotherapy dose

## Abstract

Objective: For patients with esophageal squamous cell carcinoma, preoperative chemoradiotherapy followed by planned esophagectomy is used as a curative treatment modality. However, the impact of radiotherapy dose remains undefined. Method: A total of 141 patients with stage III esophageal squamous cell carcinoma (ESCC; as defined by the 7th American Joint Committee on Cancer), receiving preoperative chemoradiotherapy followed by esophagectomy between 2000 and 2015 at Kaohsiung Chang Gung Memorial Hospital, Taiwan, were retrospectively reviewed. The radiotherapy dose of preoperative chemoradiotherapy (36 Gy before 2009 and 50–50.4 Gy after 2009) and other clinicopathological parameters were collected and correlated with the response to chemoradiotherapy and treatment outcome. Result: Of these 141 patients, the radiotherapy dose was 36 Gy in 59 (42%) patients and 50 Gy in 82 (58%) patients. A complete pathological response was noted in 12 (20%) of 59 patients receiving 36 Gy radiotherapy, and 37 (45%) of 82 patients receiving 50 Gy radiotherapy (*p* = 0.002). The three-year overall survival and disease-free survival rates were 31% and 25% in patients receiving 36 Gy radiotherapy, and 54% and 46% in patients receiving 50–50.4 Gy radiotherapy, respectively (*p* = 0.023 for overall survival; *p* = 0.047 for disease-free survival). Multivariate analysis showed that a higher radiotherapy dose was associated with increased pathological complete response (*p* = 0.003, hazard ratio: 3.215), better overall survival (*p* = 0.024, hazard ratio: 1.585), and superior disease-free survival (*p* = 0.049, hazard ratio: 1.493). However, higher radiotherapy doses revealed more surgical complications, including acute respiratory distress syndrome (*p* = 0.048) and anastomosis leaks (*p* = 0.004). Conclusion: For patients with locally advanced ESCC, preoperative chemoradiotherapy with higher radiotherapy doses led to increased pathologic complete response rates and improved survival.

## 1. Introduction

Compared to esophageal adenocarcinoma, which is more common in Western countries [1], ninety percent of esophagus malignancies in Asian countries such as Taiwan are esophageal squamous cell carcinoma (ESCC). In Taiwan, locally advanced disease is the most common condition in newly diagnosed patients with ESCC, owing to delayed diagnosis [2].

For patients with ESCC, esophagectomy plus the dissection of lymph nodes is one of the gold standard treatment modalities for curative intent. However, patients who are diagnosed with locally advanced ESCC receiving operation alone have unsatisfactory outcomes, with five-year survival below thirty percent [3,4,5,6,7]. A multimodality approach, preoperative chemoradiation therapy followed by surgery, has been advocated to downstage the primary tumor, thus increasing resectability rates and reducing micrometastases for a better survival rate [8,9,10]. The first randomized controlled trial of esophageal cancer treated with preoperative chemoradiotherapy was reported in 1992 by Nygaard et al. [11], and showed that preoperative chemoradiation therapy prolonged overall patient survival. After that literature, several studies [4,5,12,13] comparing preoperative chemoradiation therapy followed by surgery with surgery alone have shown better survival for preoperative chemoradiotherapy, whereas others have not revealed survival benefits from preoperative chemoradiotherapy compared to surgery alone [6,14,15,16,17]. In 2007, Gebski et al. conducted a meta-analysis and showed that significantly better survival was evident for preoperative chemoradiation therapy in patients with ESCC [18]. In addition, a recent phase III clinical trial [19], the Chemoradiotherapy for Oesophageal Cancer Followed by Surgery Study (CROSS), demonstrated that preoperative chemoradiotherapy has a significant survival benefit compared to surgery alone. Thus, preoperative chemoradiation therapy followed by surgery has been applied to clinical practice in many hospitals for patients with locally advanced ESCC.

Although the survival benefit has been found in preoperative chemoradiotherapy approaches, there was still a discrepancy in radiotherapy doses in preoperative chemoradiotherapy between several trials. To the best of our knowledge, a study addressing the role of radiotherapy dose of preoperative chemoradiotherapy in patients with locally advanced ESCC is lacking. Diverse radiotherapy doses of preoperative chemoradiotherapy may result in different treatment outcomes and surgical complications. Therefore, the aim of this paper is to review our experience of 141 AJCC 7th stage III ESCC patients receiving preoperative chemoradiation therapy followed by surgery and to evaluate the impact of radiotherapy doses of preoperative chemoradiotherapy on patient treatment outcomes.

## 2. Materials and Methods

### 2.1. Patient Population

Patients diagnosed esophageal squamous cell carcinoma who received preoperative chemoradiotherapy followed by surgery from 2000 to 2015 at Kaohsiung Chang Gung Memorial Hospital were reviewed retrospectively. This study was approved by the institutional review board of Chang Gung Memorial Hospital (IRB number: 202100014B0). Written informed consent was waived due to the retrospective design.

In our study, patients were evaluated by a multidisciplinary team including a thoracic surgeon, a medical oncologist, a radiation oncologist, a radiologist, and a gastroenterologist. Staging evaluation before treatment included physical examination, endoscopy, contrast-enhanced computed tomography (CT) scans from the neck to upper abdomen, positron emission tomography/computed tomography (PET-CT) scan, and/or endoscopic ultrasound (EUS). The tumor node metastasis stage (TNM) was evaluated according to the 7th American Joint Committee on Cancer (AJCC) staging system.

### 2.2. Concurrent Chemoradiotherapy Planning

Patients received treatment with two cycles of cisplatin and 5-fluorouracil-based chemotherapy and radiotherapy, concurrently. Chemotherapy including cisplatin (75 mg/m^2^; 4 h drip) on day 1 and 5-fluorouracil (1000 mg/m^2^; continuous infusion) on days 1–4 was given every 4 weeks. Radiotherapy was delivered in five daily fractions per week. Three-dimensional conformal radiotherapy (CRT) via a four-field technique or intensity-modulated radiotherapy (IMRT) with 6 or 10 MV photons was used. The gross target volume (GTV) was defined as the gross tumor and gross lymph nodes on CT scan and/or PET-CT images. The clinical target volume (CTV) comprehensively covered the esophagus, the mediastinal lymph nodes, bilateral neck, and supraclavicular lymph nodes. The planning target volume (PTV) was expended from the CTVs with a 0.5–1.0 cm margin in all directions. Before 2009, the radiotherapy total dose to the PTV was 36 Gy in 20 daily fractions administered 5 days per week. The pathological complete response rate was 20% only. Besides, our esophageal cancer team found that some patients may retract planned esophagectomy after 36 Gy preoperative chemoradiotherapy, especially for patients with drastically improved dysphagia and good response after preoperative chemoradiotherapy. These patients usually came back to our clinics with dysphagia again and even inoperable disease, because the lower preoperative radiotherapy dose of 36 Gy was not adequate to control esophageal cancer. In 2008, Tepper et al. [5] demonstrated high pathological complete response rates (pathological complete response rate 40%) after preoperative chemoradiotherapy with 50.4 Gy. After 2009, to achieve higher pathological complete response rates and adequate radiotherapy doses if patients retracted planned esophagectomy, the radiotherapy total dose to the PTV was modified to 50–50.4 Gy/25–28 fractions by our multidisciplinary team. Within 3–4 weeks following the end of irradiation, CT from the neck to upper abdomen, endoscope, and/or PET-CT scan were performed to see the treatment response. Then, the multidisciplinary team reviewed the clinical information to determine if the lesions were resectable. If lesions were classified as resectable, surgery was advised 6–12 weeks after the end of chemoradiotherapy. Patients receiving surgery had a minimally invasive esophagectomy with cervical esophagogastrostomy or an Ivor Lewis esophagectomy with intrathoracic anastomosis, two-field lymphadenectomy, and reconstruction of the gastrointestinal tract with gastric tube. Pathological complete response was defined as the complete remission of all viable cancer cells in all specimens from surgery, including the primary esophageal tumor and lymph nodes. Overall survival (OS) and disease-free survival (DFS) were defined as previously described [7].

### 2.3. Statistical Analysis

Statistical analysis was conducted with the Statistical Package for the Social Sciences (SPSS, ver. 13.0, Chicago, IL, USA). The *χ*^2^ test or Fisher’s exact test were used to compare the two groups. Multivariate analysis of pathological complete response was performed by logistic regression model. For univariate survival analysis, the Kaplan–Meier method was used, and the difference between survival curves was tested by a log-rank test. In a stepwise forward fashion, parameters with *p*-values < 0.1 at univariate level were entered into a Cox regression model to analyze their relative prognostic significance. For all analyses, two-sided tests were used with *p* < 0.05 considered significant.

## 3. Results

### 3.1. Patient Characteristics

The baseline characteristics of patients are listed in Table 1 and their distributions in radiotherapy doses of 36 Gy and 50–50.4 Gy are summarized in Table 2. At the time of analysis, the median periods of follow-up of patients receiving 36 Gy preoperative chemoradiotherapy were 156.9 months (range, 139.9–197.7 months) for the 12 survivors and 14 months (range, 3.8–197.7 months) for all 59 patients. The median periods of follow-up of patients receiving 50–50.4 Gy preoperative chemoradiotherapy were 58.3 months (range, 39.6–117 months) for the 30 survivors and 38.1 months (range, 4.5–117 months) for all 82 patients. There were no significant differences between the two groups in terms of age, sex, clinical 7th AJCC stage, clinical T classification, tumor grade, and primary tumor location. However, patients receiving 50–50.4 Gy radiotherapy dose had significantly (*p* = 0.002) more clinical N classification, N2/3, than those receiving 36 Gy radiotherapy dose. Among these 141 patients receiving esophagectomy, R0 resection was found in 121 patients, including 52 patients in the 36 Gy group and 69 patients in the 50–50.4 Gy group. R1 resection was found in 10 patients, including 6 patients in 36 Gy and 4 patients in 50–50.4 Gy groups. R2 resection was noted in 10 patients, including 1 patient in the 36 Gy group and 9 patients in the 50–50.4 Gy group.

Among these 141 patients, there were 66 (47%) patients with recurrence, including 25 (18%) patients with locoregional recurrence, 32 (23%) patients with distant recurrence, and 9 (6%) patients with locoregional and distant recurrence simultaneously. Among 59 patients receiving 36 Gy preoperative chemoradiotherapy, there were 35 (59%) patients with recurrence, including 17 (29%) patients with locoregional recurrence, 10 (17%) patients with distant recurrence, and 8 (13%) patients with locoregional and distant recurrence simultaneously. Among 82 patients receiving 50–50.4 Gy preoperative chemoradiotherapy, there were 31 (38%) patients with recurrence, including 10 (12%) patients with locoregional recurrence, 15 (18%) patients with distant recurrence, and 6 (7%) patients with locoregional and distant recurrence simultaneously. Locoregional recurrence was noted in 25 (42%) of 59 patients receiving 36 Gy preoperative chemoradiotherapy and 16 (20%) of 82 patients receiving 50–50.4 Gy preoperative chemoradiotherapy (42% versus 20%, respectively; *p* = 0.003). Distant recurrence was noted in 18 (30%) of 59 patients receiving 36 Gy preoperative chemoradiotherapy and 21 (26%) of 82 patients receiving 50–50.4 Gy preoperative chemoradiotherapy (30% versus 26%; *p* = 0.52).

### 3.2. Correlation between Clinicopathologic Parameters and Pathologic Complete Response

The relationship between the clinicopathologic parameters and pathological complete response was summarized in Table 3. We did not observe any significant correlation between pathologic complete response with age, clinical 7th AJCC stage, clinical T classification, clinical N classification, tumor grade, and primary tumor location. However, a pathological compete response was noted in 12 (20%) of 59 patients receiving 36 Gy radiotherapy, and 37 (45%) of 82 patients receiving 50–50.4 Gy radiation. Patients receiving 50–50.4 Gy radiotherapy had significantly (*p* = 0.002) higher pathological complete response rate than those receiving 36 Gy radiotherapy. The logistic model revealed that a higher radiotherapy dose (50–50.4 Gy versus 36 Gy; *p* = 0.003, hazard ratio: 3.215, 95% confidence interval: 1.493–6.944) was independently associated with increased pathological complete response after preoperative chemoradiotherapy.

### 3.3. Survival Analyses of All 141 Patients

**Figure 1:**
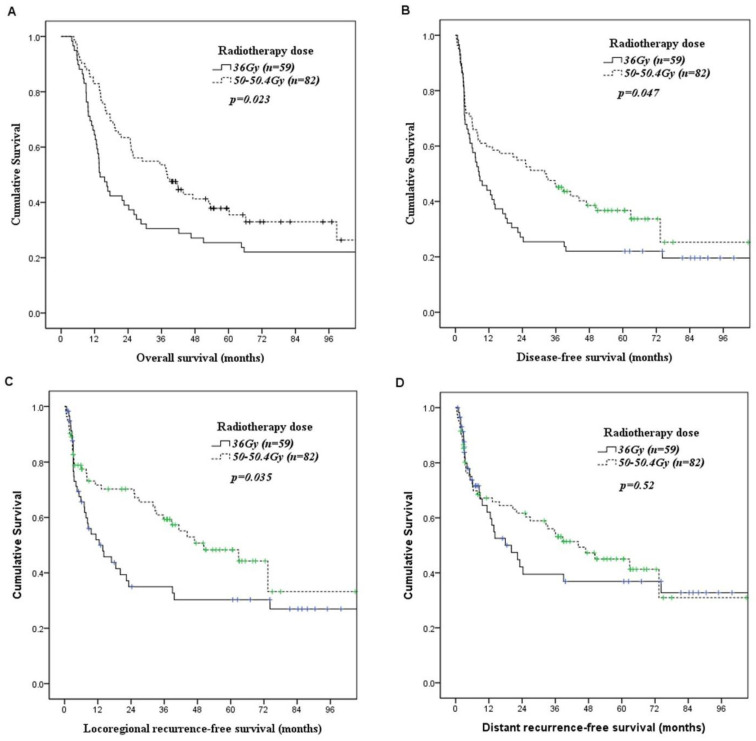
Kaplan–Meier curves according to the radiotherapy dose of preoperative chemoradiotherapy. (**A**) Overall survival. (**B**) Disease-free survival. (**C**) Locoregional recurrence-free survival. (**D**) Distant recurrence-free survival.

Correlations of preoperative chemoradiotherapy parameters with OS and DFS are summarized in Table 4. Univariate analyses found that patients receiving 50–50.4 Gy had significantly superior overall survival (*p* = 0.023, Figure 1A) and disease-free survival (*p* = 0.047, Figure 1B) than those receiving 36 Gy. Patients receiving 36 Gy preoperative chemoradiotherapy had significantly (*p* = 0.035, Figure 1C) worse locoregional recurrence-free survival than those receiving 50–50.4 Gy preoperative chemoradiotherapy. However, there was no significant difference (*p* = 0.52, Figure 1D) in distant recurrence-free survival between patients receiving 36 Gy preoperative chemoradiotherapy and patients receiving 50–50.4 Gy preoperative chemoradiotherapy. In multivariate analysis, a higher radiotherapy dose was associated with superior overall survival (50–50.4 Gy versus 36 Gy; *p* = 0.024, hazard ratio: 1.585, 95% confidence interval: 1.062–2.364, Table 5) and disease-free survival (50–50.4 Gy versus 36 Gy; *p* = 0.049, hazard ratio: 1.493, 95% confidence interval: 1.002–2.22, Table 5). The three-year overall survival and disease-free survival rates were 31% and 25% in patients receiving radiotherapy dose 36 Gy, and 54% and 46% in in patients receiving radiotherapy dose 50–50.4 Gy, respectively.

### 3.4. Analysis of Surgical Complication in 141 Patients Receiving Preoperative Chemoradiotherapy

We also performed an analysis of surgical complications in 141 patients who received esophagectomy after preoperative chemoradiotherapy. The analysis is summarized in Table 6. Higher radiotherapy doses (50–50.4 Gy) revealed significantly more acute respiratory distress syndromes and anastomosis leaks. The acute respiratory distress syndrome was noted in 3% patients receiving radiotherapy dose 36 Gy and 13% patients receiving radiotherapy dose 50–50.4 Gy (*p* = 0.042). The rates of anastomosis leak were 5% in patients receiving radiotherapy dose 36 Gy, and 23% in patients receiving radiotherapy dose 50–50.4 Gy (*p* = 0.004). There were no significant differences between two groups in terms of pneumonia, empyema, vocal cord paralysis, tracheal laceration, chylothorax, wound infection, pneumothorax, mediastinal abscess, sepsis, 30 day mortality, hospital mortality, hospital stay, and intensive care unit stay.

## 4. Discussion

In our study, we found that the three-year overall survival rates were 31% and 54% in patients receiving preoperative chemoradiotherapy with radiotherapy dose 36 Gy and 50–50.4 Gy, respectively (*p* = 0.023). Patients receiving higher radiotherapy doses had better overall survival than those receiving lower radiotherapy dose. We suggest that the survival benefit may be ascribed to more patients with pathological complete response after higher radiotherapy dose. Our study found that 12 of the 59 patients with 7th AJCC stage III ESCC receiving 36 Gy preoperative chemoradiotherapy achieved pathological complete response, a pathological compete response rate of only 20%. In the study of Bedenne et al. [9], pathological complete response was found in 25 (23%) of 110 patients with 6th AJCC T3N0-1M0 esophageal cancer receiving 30 Gy preoperative chemoradiotherapy. Fujita et al. [13] reported that the pathological complete response rate was 13% in patients with 6th AJCC T4N0-1M0 ESCC receiving 36 Gy preoperative chemoradiotherapy. Burmeister et al. [14] showed that a pathological complete response was found in 10 (27%) of 37 patients with 6th AJCC T1-3N0-1M0 ESCC receiving 35 Gy preoperative chemoradiotherapy. However, when we applied 50–50.4 Gy preoperative chemoradiotherapy, a pathological complete response was found in 37 (45%) of 82 patients. Hagen et al. [19] revealed that pathological complete response was found in 18 (49%) of 37 patients with 6th AJCC T1N1M0 or T2-3N0-1M0 ESCC receiving 41.4 Gy preoperative chemoradiotherapy. Lee et al. [15] reported a 43% pathological complete response rate in 35 patients with 6th AJCC stage II or III ESCC receiving 45.6 Gy preoperative chemoradiotherapy. In the study of Tepper et al. [5], 10 (40%) of 25 patients with 6th AJCC T1-3NxM0 esophageal cancer receiving 50.4 Gy preoperative chemoradiotherapy achieved a pathological complete response. Stahl et al. [8] showed that pathological complete response was found in 18 (32%) of 57 patients with 6th AJCC T3-4N0-1M0 ESCC receiving 40 Gy preoperative chemoradiotherapy. Yang et al. described a 43% pathological complete response rate in 185 patients with 6th AJCC T1-4N1M0/T4N0M0 receiving 40 Gy preoperative chemoradiotherapy. The above series (summarized in Table 7) seem to further support that higher radiotherapy doses of preoperative chemoradiotherapy contribute to increased pathological complete response. However, in a meta-analysis by Engel et al. [20], evaluating radiotherapy dose in patients with esophageal cancer treated by preoperative chemoradiotherapy, no difference in OS was revealed between high dose radiotherapy (>48.85 Gy biologically effective dose) and low dose radiotherapy (<48.85 Gy biologically effective dose). Further prospective study is necessary to clarify this issue.

In our study, we found that the incidence of acute respiratory distress syndrome after esophagectomy in patients receiving radiotherapy doses of 50–50.4 Gy was significantly higher (13% versus 3%) than that in patients receiving radiotherapy doses of 36 Gy. Pulmonary complications including pneumonia, acute respiratory distress syndrome, or empyema were noted in 27% of patients receiving a radiotherapy dose of 36 Gy, and 37% of patients receiving radiotherapy doses of 50–50.4 Gy. Thomas et al. [22] reported that mean lung radiation dose was a predictor for pulmonary complications in patients with esophageal cancer receiving preoperative chemoradiotherapy followed by esophagectomy. A previous study by Burmeister et al. [14] found a 20% major pulmonary complication rate in patients with 6th AJCC T1-3N0-1M0 esophageal cancer receiving 35 Gy preoperative chemoradiotherapy. However, in patients with 6th AJCC T1N1M0 or T2-3N0-1M0 esophageal cancer receiving 41.4Gy preoperative chemoradiotherapy, Hagen et al. [19] reported a 46% pulmonary complication rate. Moreover, in patients with 6th AJCC T1-3NxM0 esophageal cancer receiving 50.4 Gy preoperative chemoradiotherapy, Tepper et al. [5] described a 38% pulmonary complication rate. Among these studies, pulmonary complications were still the most common form of postoperative morbidity and seemed increase with radiation dose. Reducing the incidence of severe postoperative pulmonary complications is important in patients receiving preoperative chemoradiotherapy followed by esophagectomy to achieve better prognoses.

The rate of anastomosis leakage ranged from 3% to 30% and varied between studies. Multiple factors including radiotherapy field, radiotherapy dose, surgical procedure, and chemotherapy regimen may influence anastomosis leakage rates. Koëter et al. [23] analyzed 53 patients receiving preoperative chemoradiotherapy (41.4 Gy in 18 fractions combined with paclitaxel and carboplatin) followed by esophagectomy with cervical anastomosis, and reported that radiation dose has no impact on anastomosis leakage occurrence. However, forty-nine (92%) of 53 patients in their study had adenocarcinoma, which usually involves the lower third of the esophagus and neck area, which is the future anastomosis site and is seldom covered by radiotherapy in lower third esophageal cancer. Walle et al. [24] analyzed 54 patients receiving preoperative chemoradiotherapy (36 Gy in 20 fractions combined with cisplatin and 5-fluorouracil) followed by Ivor Lewis esophagectomy with intrathoracic anastomosis and found that the incidence of anastomosis leak was related to the radiotherapy dose on the gastric fundus. In our study, patients receiving radiotherapy doses of 50–50.4 Gy had a higher anastomosis leak rate (23% versus 5%, *p* = 0.004) than those receiving a radiotherapy dose of 36 Gy. We suggest that it may be ascribed to our radiotherapy field containing a future cervical anastomosis site. In our concurrent chemoradiotherapy planning, the esophagus, neck, and supraclavicular areas, which were future cervical anastomosis sites, were covered in the clinical target volume (CTV) of radiotherapy, and therefore higher radiotherapy doses contributed to more anastomosis leaks. Further studies with detailed radiotherapy fields, radiotherapy doses, surgical procedures, and chemotherapy regimen analysis are necessary to define the impact of radiotherapy on the incidence of anastomosis leak.

It is well known that perioperative complications have a negative impact on both OS and DFS [25,26]. In our study, although patients receiving 50–50.4 Gy preoperative chemoradiotherapy had more acute respiratory distress syndrome and anastomosis leaks than those receiving 36 Gy preoperative chemoradiotherapy, they had better OS and DFS, which is contrary to the existing literature studying perioperative complications of esophagectomy. This may be ascribed to a higher pathological complete response rate in patients receiving 50–50.4 Gy preoperative chemoradiotherapy.

Our study has important limitations. First, our results are based on a retrospective analysis which was conducted in a single hospital. The retrospective design of this analysis further justifies the conclusion that a prospective study in the future is needed to define our findings. Secondly, the patient number was small. Thirdly, the long treatment period of 15 years in our study is also an important limitation. The treatment characteristics may have changed over time. For example, PET-CT was not routinely reimbursed before 2010 by Taiwan’s health-insurance system, and thus some patients before 2010 may have been understaged. Three different surgeons joined our group in the fifteen years. Although all of them performed minimally invasive esophagectomy in the same fashion, different surgeons will lead to different results and impact the survival.

In conclusion, for patients with locally advanced esophageal squamous cell carcinoma, preoperative chemoradiotherapy with higher radiotherapy dose led to increased pathologic complete response rates and improved overall survival. In the future, prospective clinical trials evaluating the role of radiotherapy doses in patients with locally advanced esophageal squamous cell carcinoma receiving preoperative chemoradiotherapy are required.

## Figures and Tables

**Table 1:** Clinicopathologic features of 141 patients with 7th AJCC stage III esophageal squamous cell carcinoma receiving preoperative chemoradiotherapy.

Parameters	No. of Cases (Percentage)
Age (years) (mean: 52.8, median: 52, range 36–77)	
Clinical 7th AJCC stage	
IIIA	36 (25.5)
IIIB	23 (16.3)
IIIC	82 (58.2)
Clinical T classification	
T2	3 (2.1)
T3	65 (46.1)
T4	73 (51.8)
Clinical N classification	
N0	4 (2.8)
N1	65 (46.1)
N2	51 (36.2)
N3	21 (14.9)
Tumor grade	
Grade 1	23 (16.3)
Grade 2	86 (61.0)
Grade 3	32 (22.7)
Primary tumor location	
Upper	30 (21.3)
Middle	60 (42.5)
Lower	51 (36.2)
Radiotherapy dose	
36 Gy	59 (41.8)
50–50.4 Gy	82 (58.2)
Pathological complete response	
Absent	92 (65.2)
Present	49 (34.8)

**Table 2:** Associations between radiotherapy dose and clinicopathologic parameters in 141 patients with 7th AJCC stage III esophageal squamous cell carcinoma receiving preoperative chemoradiotherapy.

Parameters			Radiotherapy Dose		
			36 Gy	50–50.4 Gy	*p*-Value
Age (Mean ± Std. Deviation)			52.6 ± 8.6	52.9 ± 7.3	0.81
Sex		Male	57	79	0.93
		Female	2	3	
Clinical 7th AJCC stage		IIIA/IIIB	24	35	0.81
		IIIC	35	47	
Clinical T classification		T2/3	24	44	0.13
		T4	35	38	
Clinical N classification		N0/1	38	31	0.002 *
		N2/3	21	51	
Tumor grade		1 + 2	42	67	0.14
		3	17	15	
Primary tumor location		Upper	14	16	0.55
		Middle/Lower	45	66	
Primary tumor location		Upper/Middle	37	53	0.82
		Lower	22	29	
Locoregional recurrence		Absent	34	66	0.003 *
		Present	25	16	
Distant recurrence		Absent	41	61	0.52
		Present	18	21	

*χ*^2^ test, Fisher’s exact test, or *t*-test was used for statistical analysis. * Statistically significant.

**Table 3:** Associations between pathological complete response and clinicopathologic parameters in 141 patients with 7th AJCC stage III esophageal squamous cell carcinoma receiving preoperative chemoradiotherapy.

Parameters		Pathological Complete Response		
		Present	Absent	*p*-Value
Age	<52 y/o	17	46	0.082
	≥52 y/o	32	46	
Clinical 7th AJCC stage	IIIA/IIIB	21	38	0.86
	IIIC	28	54	
Clinical T classification	T2/3	24	44	0.90
	T4	25	48	
Clinical N classification	N0/1	26	43	0.48
	N2/3	23	49	
Tumor grade	Grade 1/2	39	70	0.64
	Grade 3	10	22	
Primary tumor location	Upper	11	19	0.80
	Middle/Lower	38	73	
Primary tumor location	Upper/Middle	32	58	0.79
	Lower	17	34	
Radiotherapy dose	36 Gy	12 (20%)	47	0.002 *
	50–50.4 Gy	37 (45%)	45	

* Statistically significant. *χ*^2^ test or Fisher’s exact test was used for statistical analysis.

**Table 4:** Results of univariate analysis of prognostic factors for overall survival and disease-free survival in 141 patients with 7th AJCC stage III esophageal squamous cell carcinoma receiving preoperative chemoradiotherapy.

Factors	No. of Patients	Overall Survival (OS)		Disease-Free Survival (DFS)	
		3-Year OS Rate (%)	*p*-Value	3-Year DFS Rate (%)	*p*-Value
Age					
<52 y/o	63	41%	0.71	35%	0.85
≥52 y/o	78	46%		40%	
Clinical 7th AJCC stage					
IIIA/IIIB	59	53%	0.14	44%	0.14
IIIC	82	38%		33%	
Clinical T classification					
T2/3	68	54%	0.057	46%	0.053
T4	73	34%		30%	
Clinical N classification					
N0/1	69	45%	0.64	35%	0.55
N2/3	72	43%		40%	
Tumor grade					
Grade 1/2	109	45%	0.85	37%	0.95
Grade 3	32	41%		41%	
Primary tumor location					
Upper	30	47%	0.84	43%	0.89
Middle/Lower	111	43%		36%	
Primary tumor location					
Upper/Middle	90	47%	0.13	42%	0.12
Lower	51	39%		31%	
Radiotherapy dose					
36 Gy	59	31%	0.023 *	25%	0.047 *
50–50.4 Gy	82	54%		46%	
Pathological complete response					
Absent	92	34%	<0.001 *	24%	<0.001 *
Present	49	63%		63%	

* Statistically significant.

**Table 5:** Results of multivariate Cox regression analysis for overall survival and disease-free survival in 141 patients with 7th AJCC stage III esophageal squamous cell carcinoma receiving preoperative chemoradiotherapy.

Factors	Overall Survival		Disease-Free Survival	
	OR (95% CI)	*p*-Value	OR (95% CI)	*p*-Value
Radiotherapy dose 50–50.4 Gy versus 36 Gy	1.585 (1.062–2.364)	0.024 *	1.493 (1.002–2.222)	0.049 *

OR, odds ratio; 95% CI, 95% confidence interval; * Statistically significant.

**Table 6:** Surgical complications and mortality after esophagectomy in 141 patients with 7th AJCC stage III esophageal squamous cell carcinoma receiving preoperative chemoradiotherapy.

Complication	36 Gy (*n* = 59)	50–50.4 Gy (*n* = 82)	*p*-Value
Pulmonary complication	16 (27%)	30 (37%)	0.24
Pneumonia	10 (17%)	18 (22%)	0.46
Acute respiratory distress syndrome (ARDS)	2 (3%)	11 (13%)	0.042 *
Empyema	4 (7%)	1 (1%)	0.072
Anastomosis leak	3 (5%)	19 (23%)	0.004 *
Vocal cord paralysis	3 (5%)	2 (2%)	0.65
Tracheal laceration	0 (0%)	1 (1%)	1.00
Chylothorax	0 (0%)	5 (6%)	0.075
Wound infection (except anastomosis)	1 (2%)	1 (1%)	1.00
Pneumothorax	1 (2%)	2 (2%)	1.00
Mediastinal abscess	0 (0%)	1 (1%)	1.00
Sepsis (and shock)	2 (3%)	2 (2%)	1.00
30 day mortality	0 (0%)	3 (4%)	0.27
Hospital mortality	4 (7%)	7 (9%)	0.76
Post-OP hospital stay	32.67	30.99	0.79
Post-OP ICU stay	9.08	7.72	0.63
Post-OP wean ventilator days	5.05	4.66	0.89

OP, operation; ICU, intensive care unit; * Statistically significant.

**Table 7:** Clinical series of patients with esophageal cancer receiving preoperative chemoradiotherapy.

Authors	Year	RT Dose	Chemotherapy Regimen	AJCC Staging	pCR
Lee et al. [15]	2004	45.6 Gy	Cisplatin/5-FU	6th stage II and III	43% (15/35 ESCC)
Fujita et al. [13]	2005	36 Gy	Cisplatin/5-FU	6th T4N0-1	13% (2/15 ESCC)
Burmeister et al. [14]	2005	35 Gy	Cisplatin/5-FU	6th T1-3N0-1	27% (10/37 ESCC)
Stahl et al. [8]	2005	40 Gy	Cisplatin/etoposide	6th T3-4N0-1	32% (18/57 ESCC)
Bedenne et al. [9]	2007	30 Gy	Cisplatin/5-FU	6th T3N0-1	23% (25/110 EC)
Tepper et al. [5]	2008	50.4 Gy	Cisplatin/5-FU	6th T1-3Nx	40% (10/25 EC)
Hagen et al. [19]	2012	41.4 Gy	Paclitaxel/carboplatin	6th T1N1 or T2-3N0-1	49% (18/37 ESCC)
Yang et al. [21]	2018	40 Gy	Cisplatin/vinorelbine	6th T1-4N1/T4N0	43% (80/185 ESCC)
Our study		36 Gy	Cisplatin/5-FU	7th stage III	20% (12/59 ESCC)
		50–50.4 Gy	Cisplatin/5-FU	7th stage III	45% (37/82 ESCC)

RT, radiotherapy; AJCC, American Joint Committee on Cancer; pCR, pathological complete response; 5-FU, 5-fluorouracil; ESCC, esophageal squamous cell carcinoma; EC, esophageal cancer.

## Data Availability

The data presented in this study are available on request from the corresponding author. The data are not publicly available due to privacy and confidentiality.

## Abbreviations

ESCC	esophageal squamous cell carcinoma
CROSS	Chemoradiotherapy for Oesophageal Cancer Followed by Surgery Study
AJCC	American Joint Committee on Cancer
CT	computed tomography
PET	positron emission tomography
EUS	endoscopic ultrasound
TNM	tumor node metastasis stage
CRT	conformal radiotherapy
IMRT	intensity-modulated radiotherapy
GTV	gross target volume
CTV	clinical target volume
PTV	planning target volume
OS	overall survival
DFS	disease-free survival
SPSS	Statistical Package for the Social Sciences
